# A Yeast GSK-3 Kinase Mck1 Promotes Cdc6 Degradation to Inhibit DNA Re-Replication

**DOI:** 10.1371/journal.pgen.1003099

**Published:** 2012-12-06

**Authors:** Amy E. Ikui, Valentina Rossio, Lea Schroeder, Satoshi Yoshida

**Affiliations:** 1 Department of Biology, Brooklyn College, The City University of New York, Brooklyn, New York, United States of America; 2 Department of Biology, Brandeis University, Waltham, Massachusetts, United States of America; The University of North Carolina at Chapel Hill, United States of America

## Abstract

Cdc6p is an essential component of the pre-replicative complex (pre-RC), which binds to DNA replication origins to promote initiation of DNA replication. Only once per cell cycle does DNA replication take place. After initiation, the pre-RC components are disassembled in order to prevent re-replication. It has been shown that the N-terminal region of Cdc6p is targeted for degradation after phosphorylation by Cyclin Dependent Kinase (CDK). Here we show that Mck1p, a yeast homologue of GSK-3 kinase, is also required for Cdc6 degradation through a distinct mechanism. Cdc6 is an unstable protein and is accumulated in the nucleus only during G1 and early S-phase in wild-type cells. In *mck1* deletion cells, CDC6p is stabilized and accumulates in the nucleus even in late S phase and mitosis. Overexpression of Mck1p induces rapid Cdc6p degradation in a manner dependent on Threonine-368, a GSK-3 phosphorylation consensus site, and SCF^CDC4^. We show evidence that Mck1p-dependent degradation of Cdc6 is required for prevention of DNA re-replication. Loss of Mck1 activity results in synthetic lethality with other pre-RC mutants previously implicated in re-replication control, and these double mutant strains over-replicate DNA within a single cell cycle. These results suggest that a GSK3 family protein plays an unexpected role in preventing DNA over-replication through Cdc6 degradation in *Saccharomyces cerevisiae*. We propose that both CDK and Mck1 kinases are required for Cdc6 degradation to ensure a tight control of DNA replication.

## Author Summary

DNA replication is a fundamental cellular process that takes place in all living organisms. This cellular event has to be tightly regulated to ensure an accurate genome integrity such that DNA replication takes place only once per cell cycle. Here we show a mechanism by which DNA re-replication is controlled by Cyclin Dependent Kinase (CDK) and a yeast GSK-3 kinase (Mck1p) in *S. cerevisiae*. We found that Mck1p promoted Cdc6 protein degradation. Mck1p targets Cdc6p through a GSK-3 consensus site (T368), and Cdc6p protein degradation was also mediated through the same T368 site. The GSK-3 kinase has diverse cellular functions in higher eukaryotes including roles in tumorigenesis. This finding is particularly important, since this is the first evidence to show that a GSK-3 family kinase regulates DNA replication.

## Introduction

To constitute the pre-RC and initiate DNA replication, all six-components of the Origin Recognition Complex (Orc1-6p) bind to replication origins followed by Cdc6p, Cdt1p and the Mcm2-7p complex [1]. Then the pre-RC has to be activated by the Dbf kinase-Cdc7p complex, resulting in the formation of a bidirectional replication fork in which the Mcm complex acts as a replicative helicase [1]. Finally, DNA polymerase synthesizes new strands of DNA. The cell cycle progression is driven by the Cyclin/CDK complex. Of the nine cyclins in *S. cerevisiae* six are B-type cyclins (Clb1-6) [2] and there is a single CDK (Cdc28). Cdc28-Clb activity is required to initiate DNA replication [3]–[5].

Eukaryotes ensure that DNA is replicated once and only once per cell cycle. There are multiple overlapping mechanisms to prevent re-initiation of DNA replication. Pre-RC components such as Cdc6, Mcm2–7, and the ORC complex are phosphorylated by Cyclin/CDK to prevent a second round of DNA replication from occurring before mitosis. Cdc6 is phosphorylated by Cyclin/CDK complex at the N-terminal region and is targeted for ubiquitin-mediated proteolysis in *S. cerevisiae* [6]–[8]. The MCM complex is translocated to the cytoplasm after phosphorylation by Cdk activity [9], [10]. Orc2 and Orc6 are also phosphorylated in a CDK-dependent manner [11], [12]. In addition to these mechanisms, a direct recruitment of the cyclin-CDK complex Clb5p-Cdc28p to the origin of replication is an important component of re-replication control [13]. The Clb5p recruitment to the origin is accomplished by binding of the Clb5p hydrophobic patch substrate-targeting domain [14]–[16] to an Arg-X-Leu (RXL) target sequence in the Orc6p subunit of the ORC origin recognition complex [13]. This Clb5 binding to Orc6 after origin licensing serves as a local switch to inhibit DNA re-replication by preventing Cdt1/Mcm2–7 loading onto the origin [17]. The *ORC6-rxl* mutation strongly synergized with other mutations previously implicated in re-replication control including: N-terminal deletions in Cdc6 which stabilize the protein (*CDC6ΔNT*) [13], mutations which force nuclear localization of the Mcm complex *(MCM7-NLS)* [11], and mutations blocking Orc2 *(ORC2-ps)* and Orc6 phosphorylation *(ORC6-ps)* [18]. Such multiple mutant strains strongly over-replicate DNA within a single cell cycle [13].

*ORC6-rxl GAL-CDC6ΔNT* cells are viable, but show moderate DNA re-replication when incubated in galactose [19]. The cell cycle in the *ORC6-rxl GAL-CDC6ΔNT* cells arrest at G2/M phase due to DNA damage checkpoint activation [19]. Moderate cell viability in the *ORC6-rxl GAL-CDC6ΔNT* cells was heavily dependent on DNA damage checkpoint components such as *MRE11* gene. Cell viability was reduced and DNA re-replication was enhanced in *mre11 ORC6-rxl GAL-CDC6ΔNT* cells [19]. It is known that Rad53 is phosphorylated upon DNA damage checkpoint activation. Rad53 was hyperphosphorylated in *ORC6-rxl GAL-CDC6ΔNT* cells [19], suggesting that DNA damage was induced. We concluded that DNA re-replication most likely causes double strand breaks which in turn activates the DNA damage checkpoint response [19].

To identify a new component that inhibits DNA re-replication in *S. cerevisiae*, synthetic genetic array (SGA analysis) [20] was performed using an *ORC6-rxl* strain to eliminate Clb5-Orc6 binding. We found that *mck1* deletion cells combined with the *ORC6-rxl* mutation showed synthetic lethality. The *MCK1* gene in *S. cerevisiae* encodes a serine/threonine protein kinase homologous to mammalian glycogen synthase kinase-3 (GSK-3) [21]. Mammalian GSK-3 was initially identified as an enzyme involved in the control of glycogen metabolism [22]. GSK-3 kinase is highly conserved through evolution and plays an important role in the Wnt signaling pathway in the mammalian system (for a review, see [23]). One of the interesting features of GSK-3 kinase is its role in protein degradation. GSK-3 phosphorylates cyclin D1 to promote its nuclear export and subsequent degradation in the mammalian system [24]. Yeast Mck1p has diverse biological functions. Mck1p stimulates calcineurin signaling [25]–[27] and binds stress-response elements to activate transcription [27] therefore cells lacking Mck1p are hot and cold sensitive [28]. Mck1 is also implicated in mitosis and meiosis. Yeast *MCK1* has been isolated as a dosage suppressor of centromere (*CEN*) DNA mutation in *CDEIII*, suggesting that Mck1 has a role in centromere/kinetochore function [28]. The *mck1* mutant exhibits poor sporulation [29], and sensitivity to benomyl, a microtubule destabilizing drug [28].

Cdc6 levels are regulated by three distinct mechanisms: transcription [30], ubiquitin-mediated proteolysis [7], [8], [31], [32] and nuclear localization [33]. Here we show that Mck1p has a novel function in inhibition of DNA re-replication by Cdc6p degradation through the GSK-3 consensus site at T368.

## Results

### Deletion of *MCK1* causes synthetic lethality in the *orc* mutants

**Figure 1:**
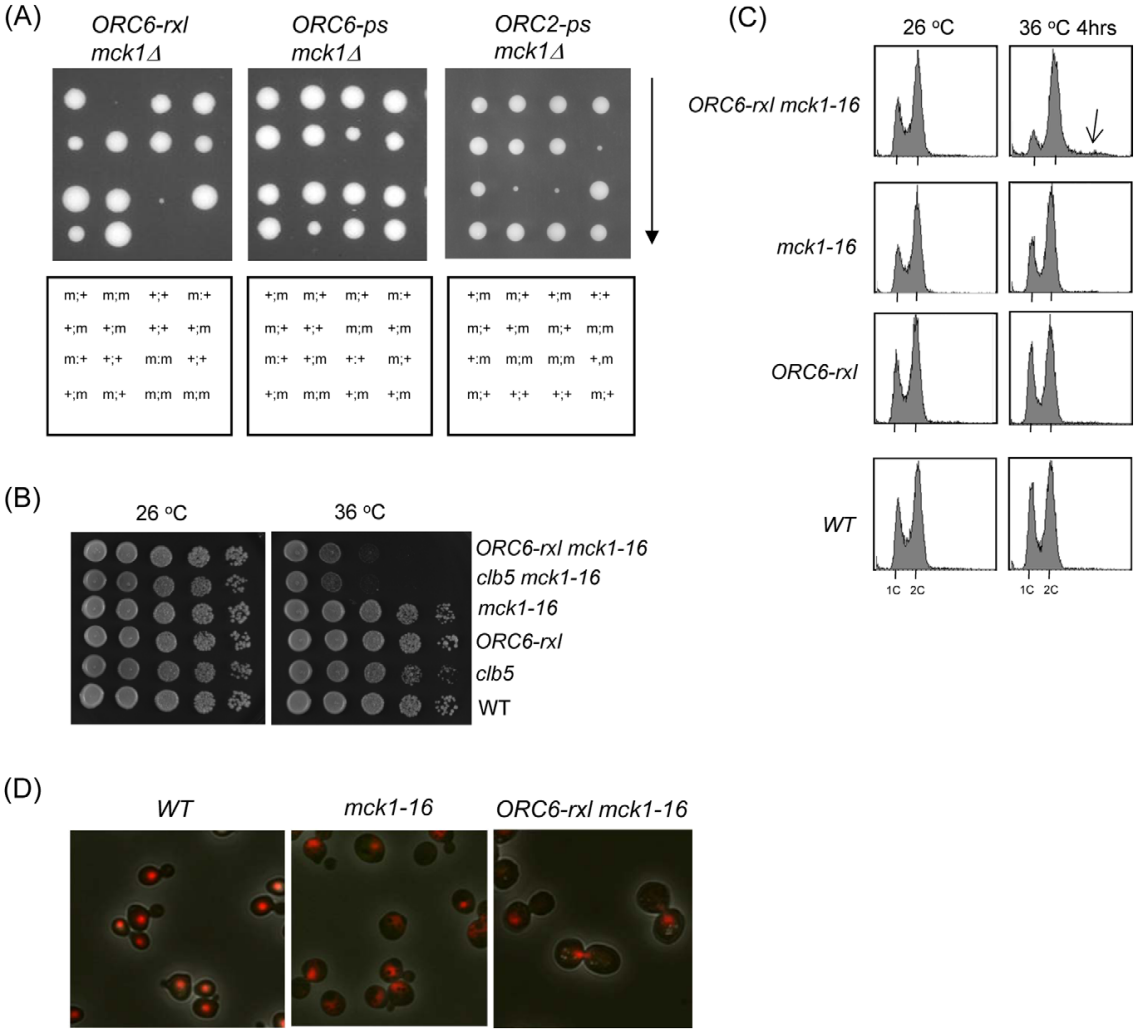
Synthetic lethality and mitotic arrest was induced in the *ORC6-rxl mck1-16* cells. (A) *ORC-x::LEU2::HIS3/ORC6 mck1/MCK1-wt* diploid strains were sporulated, tetrads were dissected on YEPD plates, and the plates were incubated for 3 days at 30°C. *ORC6* alleles and *mck1* deletion were identified based on the markers. Inviable spores were genotyped by assuming a 2∶2 segregation. The *ORC6-rxl*, *ORC6-ps* or *ORC2-ps* alleles were indicated above each panel. The presence of *ORC6* mutant allele was marked as (m) and *ORC6* wild type as (+) on the left. The presence of *mck1* mutant allele (m) or *MCK1-wt* (+) was indicated on the right. (B) Strains with indicated genotypes were serially diluted 10 fold and plated on YEPD plates to test viability at different temperatures. The plates were incubated at the indicated temperature for 1–2 days. (C) Asynchronus populations of strains with indicated genotypes were grown at 26°C first. The temperature was shifted to 36°C and the samples were collected after 4 hours. The cells were fixed, stained with propidium iodide and analyzed by FACS. (D) Wild type, *mck1-16* or *ORC6-rxl mck1-16* cells were incubated at 36°C for 4 hours, and observed under the fluorescent microscope. Red color indicates nuclei stained by propidium iodide.

Synthetic genetic array (SGA analysis) [20] was performed using *ORC6-rxl*, to eliminate Clb5-Orc6 binding, in order to identify a new component in the regulation of DNA re-replication in *S. cerevisiae*. We found that *mck1* deletion cells showed synthetic lethality in cells containing the *ORC6-rxl* mutation. It is interesting that *mck1* was the only deletion strain that caused synthetic lethality in the *ORC6-rxl* cells among 4700 deletion strains tested, and that we did not obtain other GSK-3 orthologs in this screening. Tetrad analysis confirmed the genetic interaction between *ORC6-rxl* and *mck1* deletion strains (Figure 1A). Haploid progenies, which contain both *ORC6-rxl* and *Δmck1* mutations, were not able to grow on YEPD plates whereas single mutants grew fine. We also tested if the *mck1* deletion genetically interacts with the other *orc* mutants such as the Orc6 phosphorylation site mutant (*ORC6-ps*) and the Orc2 phosphorylation site mutant (*ORC2-ps*). Deletion of *MCK1* reduced cell growth in the *ORC6-ps* cells (Figure 1A). Furthermore, the *mck1* deletion caused severe growth defects in the *ORC2-ps* cells (Figure 1A). Thus, *mck1* deletion caused synthetic lethality or semi-lethality with DNA re-replication-prone *orc* mutants in general. This strongly suggests that Mck1p has a function in DNA replication control. The *mck1* deletion strain did not have genetic interactions with other pre-RC mutants such as *MCM7-NLS* or *CDC6ΔNT* (data not shown).

### Combination of *mck1* deletion and *ORC6-rxl* mutation induced DNA damage checkpoint activation

To investigate the molecular basis of the synthetic lethality between *Δmck1* and *ORC6-rxl*, we generated partial loss of function mutants of *mck1* by PCR mutagenesis. Among them, *mck1-16* allele exhibited semi-synthetic lethality at high temperature (36 degrees) when combined with *ORC6-rxl* mutation (Figure 1B). Consistent with this effect being due to the disruption of Clb5-Orc6 protein interaction by the *ORC6-rxl* mutation, the *clb5 mck1-16* cells were also semi-lethal when incubated at 36 degrees (Figure 1B). To analyze the terminal phenotype of the *mck1-16 ORC6-rxl* strain, cells were incubated either at permissive or non-permissive temperatures and cell cycle profiles were analyzed by flow cytometry analysis. The *mck1-16 ORC6-rxl* cells showed G2/M arrest after 4 hours incubation at 36 degrees (Figure 1C, top right), with some cells showing a DNA content over 2C (Figure 1C, arrow), suggesting re-replicated DNA. Cell morphologies of the *mck1-16 ORC6-rxl* mutants were further analyzed. The *mck1-16 ORC6-rxl* cells incubated at 36 degrees for 4 hours showed large budded cells with a single nuclei visualized by propidium iodide staining of DNA (Figure 1D). This phenotype is reminiscent of cells with DNA re-replication found in our previous report [19]. Nuclear division did not occur in the *mck1-16 ORC6-rxl* cells. Their cell cycle is arrested during G2 or early mitosis, most likely due to DNA damage checkpoint activated by DNA re-replication. This is similar to our previous observation that mitotic arrest in the *ORC6-rxl CDC6ΔNT* cells was due to DNA damage [19].

**Figure 2:**
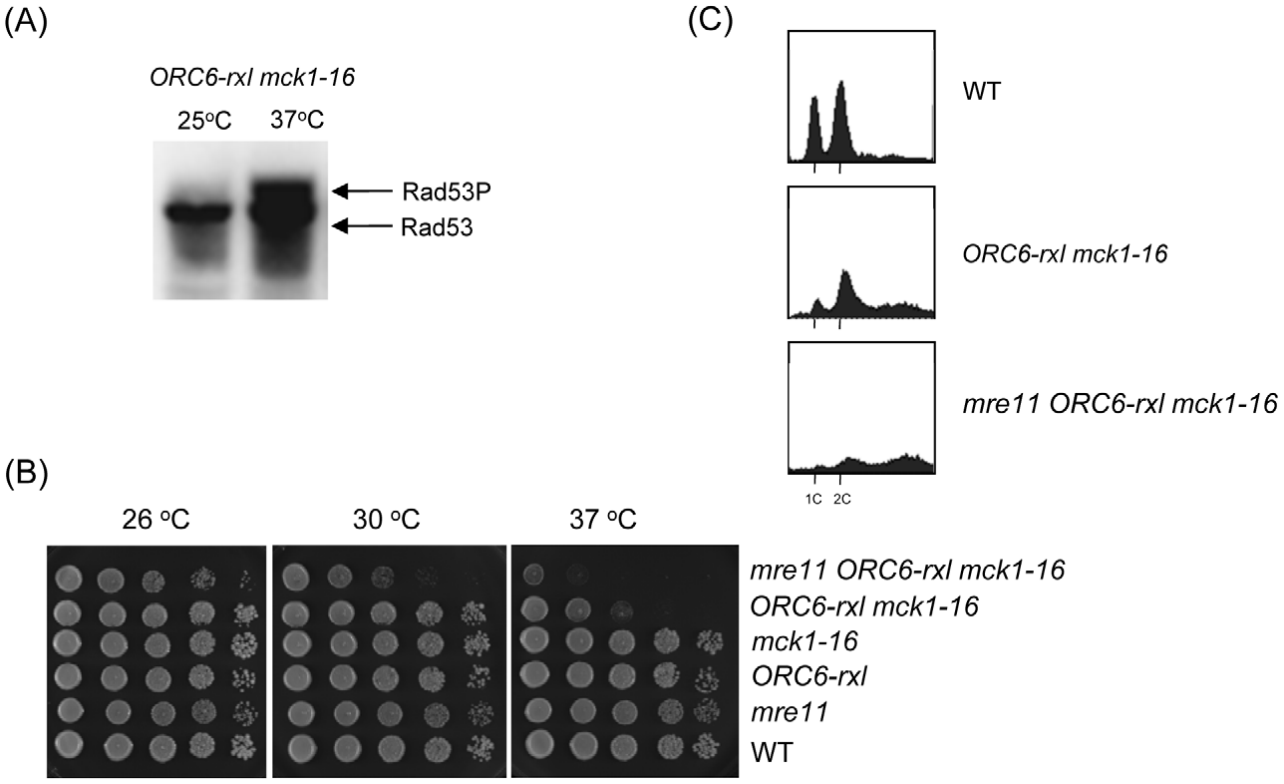
DNA damage was induced in *ORC6-rxl mck1-16* cells. (A) *ORC6-rxl mck1-16* cells were incubated either at 25 or 37°C and their protein extract was subjected to western blot analysis to detect Rad53-FLAG. (B) Strains with indicated genotypes were serially diluted 10 fold and plated on YEPD plates. The plates were incubated at the indicated temperature for 1–2 days. (C) Asynchronus populations of strains with indicated genotypes were grown at 25°C first. The temperature was shifted to 37°C for 8 hours and samples were collected. The cells were fixed, stained with propidium iodide and analyzed by FACS.

Previously we have shown that the *ORC6-rxl* mutant causes semi-synthetic lethality with a *CDC6ΔNT* mutant. The *ORC6-rxl CDC6ΔNT* cells are arrested during mitosis with moderate DNA re-replication followed by DNA damage. Viability of the *ORC6-rxl CDC6ΔNT* cells was heavily dependent on an intact DNA damage checkpoint gene such as *MRE11*, a component of the *MRX* complex [19]. Rad53, a transducer kinase required for DNA damage checkpoint activation, was hyperphosphorylated in the *ORC6-rxl CDC6ΔNT* cells. To directly test if DNA damage checkpoint is activated in the *mck-16 ORC6-rxl* cells, Rad53 phosphorylation status was analyzed by Western blotting. Rad53 was only hyperphosphorylated in the *mck-16 ORC6-rxl* cells when incubated at 37 degrees (Figure 2A). We tested if the viability of the *mck1-16 ORC6-rxl* mutant also relies on DNA damage checkpoint. We found that cell viability of the *mck-16 ORC6-rxl* cells even at the permissive temperature (30 degrees) required *MRE11* (Figure 2B). Next, the cell cycle profile of the *mre11 mck-16 ORC6-rxl* cells was examined. DNA re-replication was greatly enhanced in the *mre11 mck-16 ORC6-rxl* cells at the non-permissive temperature, indicating that DNA damage checkpoint activation limits DNA re-replication in the *mck-16 ORC6-rxl* cells (Figure 2C). Above all, we conclude that an induction of DNA re-replication in the *mck-16 ORC6-rxl* cells triggered DNA damage leading to cell cycle arrest by DNA damage checkpoint activation.

### Mck1 prevents DNA re-replication in parallel to ORC and MCM complexes

**Figure 3:**
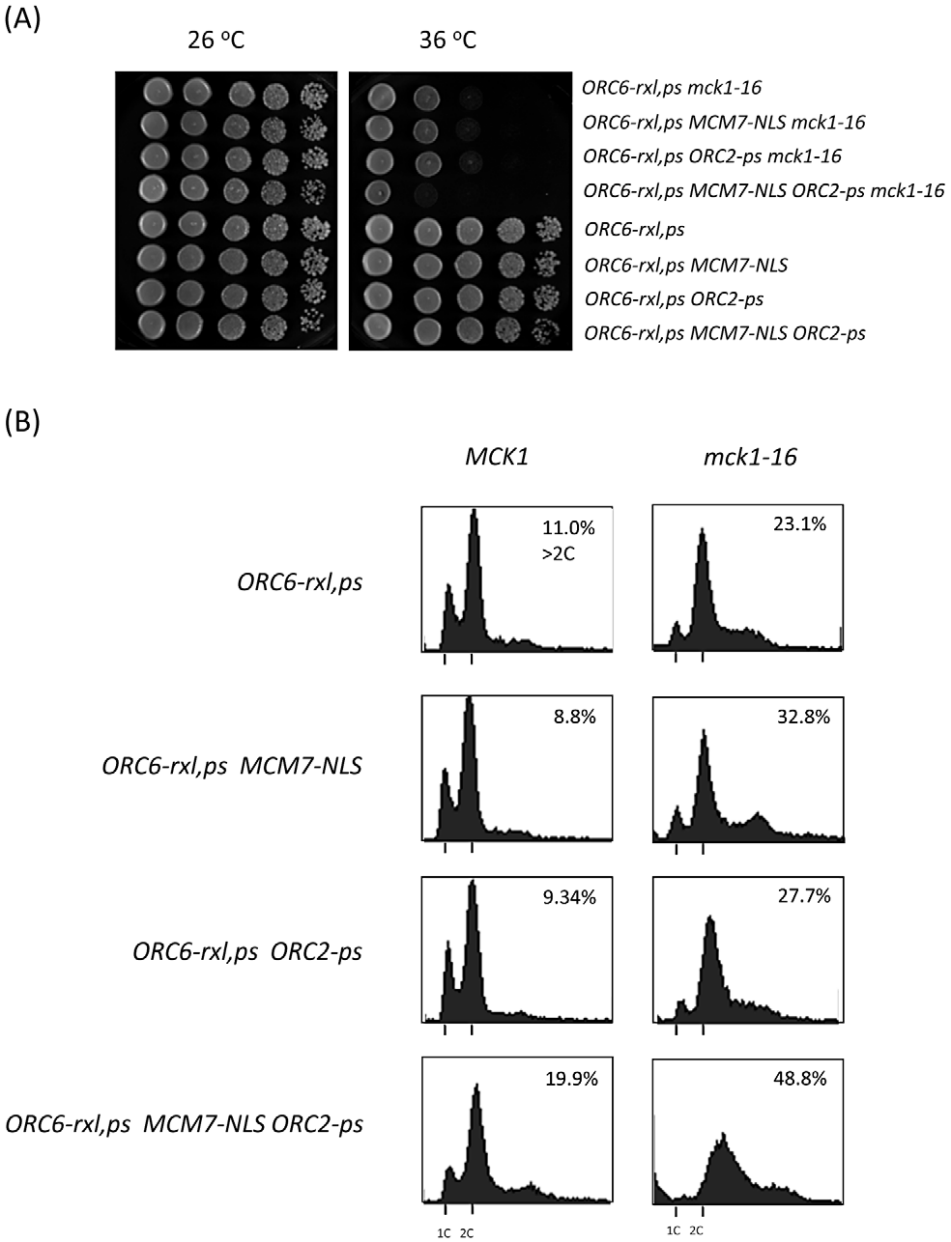
Mechanism of DNA re-replication control by Mck1 kinase is additive. (A) Cells with indicated genotypes were plated with a 10-fold dilution on YEPD plates. The plates were incubated at indicated temperatures for 2 days. (B) Asynchronus population cells with indicated genotypes (with wild type *MCK1* or with *mck1-16* mutation) were incubated at 26°C first and then shifted to 36°C for 4 hours. Cells were fixed, stained by propidium iodide and analyzed by FACS. Percentage of the cell population over 2C DNA content is shown.

Several parallel and partially overlapped molecular mechanisms ensure that cells do not re-initiate DNA replication at origins that have already fired. We have previously shown that *ORC6-rxl CDC6ΔNT* cells are mitotic arrested without extensive DNA re-replication [19]. However, multiple mutant strains such as *ORC6-rxl,ps CDC6ΔNT MCM7-NLS ORC2-ps* strongly over-replicate DNA within a single cell cycle [13]. We tested if *mck1* deletion also synergizes with other pre-RC mutations. An addition of either *MCM7-NLS* or *ORC2-ps* mutation to the *ORC6-rxl mck1-16* did not enhance lethality (Figure 3A). However, cells containing *ORC6-rxl,ps mck1-16 MCM7-NLS* and *ORC2-ps* mutations showed stronger lethality (Figure 3A). Flow cytometry analysis showed that DNA re-replication was enhanced in the *ORC6-rxl,ps mck1-16 MCM7-NLS ORC2-ps* mutant after 4 hours incubation at the non-permissive temperature (Figure 3B, bottom right). *ORC6-rxl,ps MCM7-NLS ORC2-ps* cells with wild type *MCK1* grew normally and did not induce significant re-replication (Figure 3A and 3B bottom left). These results show that Mck1p contributes to the inhibition of DNA re-replication and suggest that the mechanism involved is likely to be distinct from the known mechanisms acting at the level of ORC and MCM proteins.

### The *mck1* deletion strain genetically interacted with S-phase cyclins, but not mitotic cyclins

The semi-lethal phenotype of *ORC6-rxl Δmck1* cells (Figure 1D) was reminiscent of *ORC6-rxl CDC6ΔNT* cells [13]. Moreover, the deletion of *MCK1* interacted genetically with *ORC6-rxl* (Figure 1A) but not *CDC6ΔNT* (data not shown). These observations led us to hypothesize that Mck1p could function in DNA replication control by regulating Cdc6. To further test this model, we examined if *mck1* deletion behaved similarly to *CDC6ΔNT* in its interactions with mutations in the cyclin genes.

*CDC6ΔNT* genetically interacts with the *clb5* deletion mutant, but not with other B-type cyclins [34]. We also tested if *mck1* deletion cells genetically interact with other cyclin mutants in a similar way that *CDC6ΔNT* does. Table 1 summarizes the genetic interaction between *mck1* and cyclin mutants. The *mck1* deletion cells were semi-lethal in the *ORC6-rxl* mutant cells and also showed synthetic lethality with *clb5* deletion cells because *ORC6-rxl* is a binding mutant for Clb5p. However, the *mck1* deletion cells did not cause synthetic lethality with other B-type cyclin mutants such as *clb1*,*2*,*3*,*4* or *6* (Table 1). Therefore, *mck1* deletion genetically interacts specifically with *clb5* deletion. It has been shown that Clb5p binds to Orc6p through the Clb5p hydrophobic patch substrate-targeting domain [14]. We tested if *clb5-hpm* (Clb5 hydrophobic patch mutant) causes synthetic lethality with *Δmck1* cells and found that there was a genetic interaction between *clb5-hpm* and *Δmck1* (Table 1). Moreover neither *mck1* nor *CDC6ΔNT* caused lethality in *clb5pCLB2*, a mutant in which Clb2 is controlled under Clb5 promoter. Thus, we conclude that the *Δmck1* cells require Clb5p-Orc6p protein binding for their survival. We also found that deletion of *CLB6* rescues *Δmck1 Δclb5* semi-lethality. We have previously shown that lethality in *clb5 CDC6ΔNT* cells can be rescued by the deletion of *CLB6* [34] and proposed the idea that the S-phase cyclin Clb6 initiates DNA replication, but fails to inhibit DNA re-replication. Therefore, the DNA re-replication phenotype is suppressed if *CLB6* is deleted by the reduction of initiation of DNA replication. Mitotic cyclins regulate DNA replication in the *clb5 clb6 ORC6-rxl* cells. We speculate that deletion of *CLB6* rescues *Δmck1 Δclb5* cells in the same manner.

**Table 1:** Genetic interaction between *mck1* deletion and cyclin mutants.

	*Δmck1*	*CDC6ΔNT*
*ORC6-rxl*	sick	sick
*clb5*	lethal	lethal
*clb5 clb6*	*clb6* rescued *clb5 mck1* lethality	*clb6* rescued *clb5 CDC6ΔNT* lethality
*clb2 clb4*	viable	viable
*clb1 clb3 clb4*	viable	viable
*clb5-hpm*	sick	sick
*clb5pCLB2*	viable	viable

From these results we conclude that the *mck1* deletions genetically interacted with cyclin mutants in a way similar to that of stabilized *CDC6ΔNT*, reinforcing a model in which Mck1p acts in the same pathway as Cdc6p.

### Mck1p kinase is required for Cdc6p degradation in mitosis

**Figure 4:**
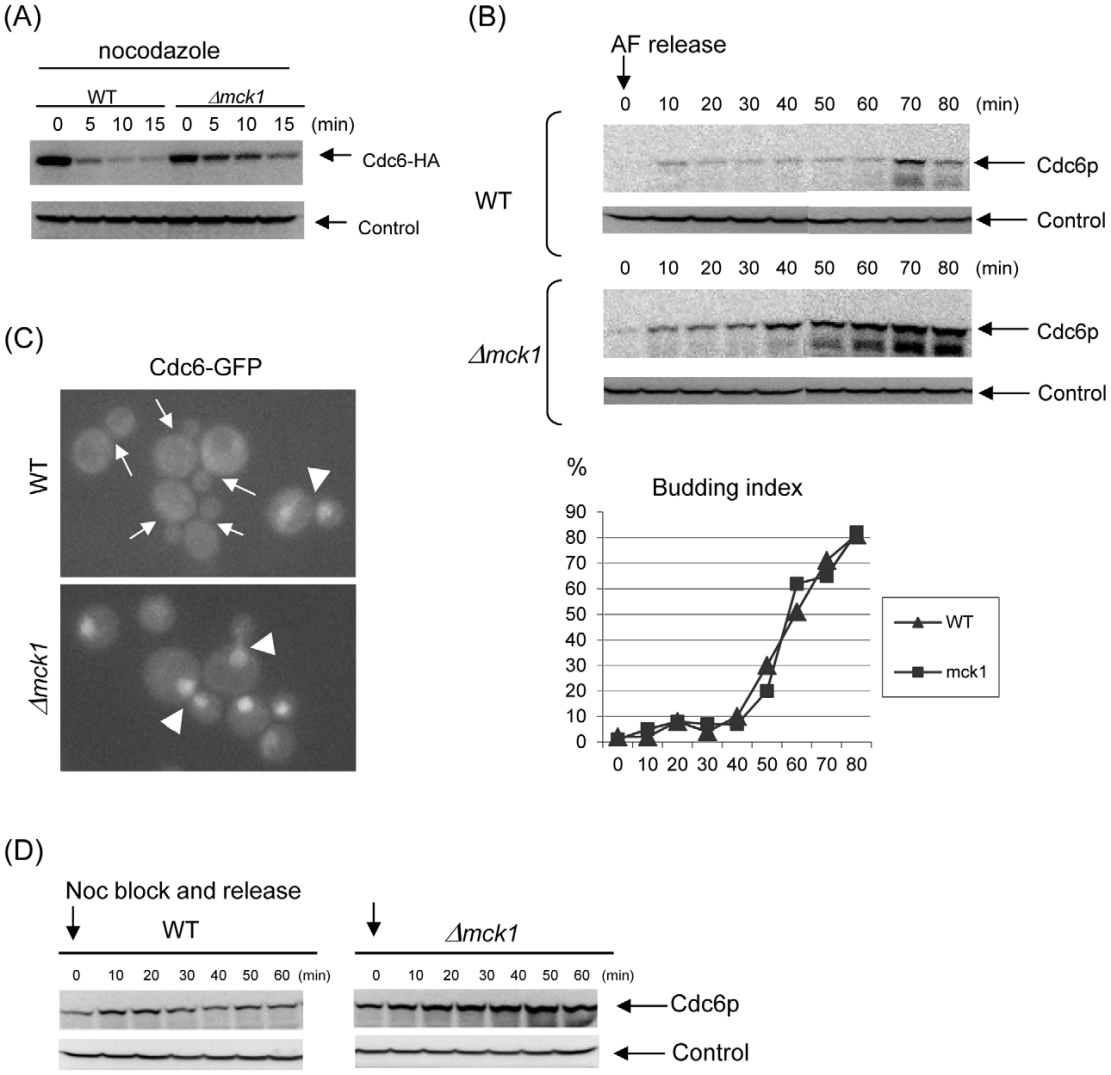
Mck1p is required for Cdc6p degradation. (A) *GAL-CDC6-HA* (WT) or *Δmck1 GAL-CDC6-HA* (*Δmck1*) cells were incubated in raffinose first, then transferred to galactose media for 2 hours. Nocodazole was added and the cells were incubated for 2 more hours. Cdc6 expression was suppressed by adding glucose. Samples were collected every 5 minutes. Protein extracts were made and subjected to western blot analysis to observe Cdc6-HA. Pgk1 was used as a loading control. (B) *CDC6-prA* or *Δmck1 CDC6-prA* cells were treated with alpha-factor to arrest the cell cycle during G1 phase. The cells were released from G1 and collected every 10 minutes. Proteins were extracted from each sample and subjected to Western blot analysis to detect Cdc6-prA. Budding index is shown using the same samples. (C) Localization of Cdc6-GFP was analyzed in living cells. In wild type cells, nuclear accumulation of Cdc6-GFP was observed only in late mitotic cells (arrowhead) and not in small to medium budded cells (arrows), whereas Cdc6-GFP was observed in the nucleus throughout the cell cycle (arrowheads) in *mck1* deletion cells. (D) *CDC6-prA* or *Δmck1 CDC6-prA* cells were blocked at metaphase by nocodazole. The mitotic arrest was released upon removal of the drug. Cells were then incubated in YEPD and collected every 10 minutes. Proteins were extracted subjected to western blot analysis to detect CDC6-prA. Pgk1 was used as a loading control.

Because lack of Mck1p and stabilization of Cdc6p (Cdc6ΔNT) exhibited similar genetic interaction with DNA re-replication mutants, we speculated that Mck1p could control the stability of Cdc6p. To test this possibility, the Cdc6 protein (Cdc6-HA) expressed under inducible *GAL1* promoter in mitotically arrested cells was examined in wild type or *Δmck1* backgrounds. We found that the Cdc6 protein level was sustained at a higher level during mitosis in the *mck1* deletion cells than in wild type cells even after Cdc6 expression was shut off by glucose (Figure 4A). It is important to mention that *CDC6* was expressed under the *GAL1* promoter, excluding possible involvement of *CDC6* transcription by Mck1 in this experiment. To test if Mck1 regulates Cdc6p post-translational levels, endogenous Cdc6 synthesis was blocked by cycloheximide. In the mitotically arrested wild type cells, Cdc6 protein was rapidly depleted by addition of cycloheximide (Figure S1). In the mitotic *mck1* deletion cells, the cdc6 protein level was high and remained stable after cycloheximide, excluding the possibility that Mck1p regulates Cdc6p by translation. These results strongly suggest that Mck1p controls Cdc6 protein levels by affecting degradation rates.

To further explore the possible involvement of Mck1p in Cdc6p degradation, Protein A-tagged Cdc6 protein integrated at the genome locus was examined in the wild type or *mck1* deletion cells by Western blotting throughout a single cell cycle progression. We noticed a dramatic accumulation of Cdc6 protein in the *mck1* deletion cells (Figure 4B). In wild type cells, Cdc6p was expressed transiently during G1 phase, 10 minutes after alpha-factor release, and suppressed throughout S-phase. Then Cdc6p was expressed again for a short time during mitosis, 70 minutes after alpha-factor release (Figure 4B, upper panel). This is consistent with a previous report by Drury et al [32]. While in the *mck1* deletion cells, Cdc6p was not expressed during alpha-factor arrest but was expressed 10 min after alpha-factor release and continued to accumulate during S-phase and mitosis (Figure 4B, lower panel). The increase in Cdc6 protein level is unlikely to be due to an alteration in the cell cycle progression of *Δmck1* cells because the kinetics of the cell cycle progression was similar in these two strains as judged by budding index (Figure 4B). To confirm that Cdc6p is stabilized during mitosis in the *mck1* deletion strain, *CDC6-ProteinA* or *mck1 CDC6-ProteinA* strains were arrested in mitosis by nocodazole and were synchronously released into the cell cycle by washing. A small amount of Cdc6p was detectable at time zero in nocodazole arrested wild type cells (Figure 4D, left). This amount was transiently increased 10–20 minutes after release. This is consistent with a previous report that Cdc6 protein is expressed in late mitosis and degraded after the G1/S transition [7]. In contrast, Cdc6p was stabilized throughout mitotic progression in the *mck1* deletion cells (Figure 4D, right).

To further confirm if Cdc6 is stabilized in the *mck1* deletion cells, we visualized Cdc6p localization *in vivo*. We introduced a GFP-tag into the C-terminus of the chromosomal copy of the *CDC6* gene to allow endogenous expression. The *CDC6-GFP* fusion appears to be fully functional as a *CDC6-GFP* strain and did not show any growth defect in any of the conditions tested (data not shown). Consistent with previously published localization patterns of overexpression, Cdc6-GFP [33], [35] protein localized and accumulated in the nucleus in late mitotic cells (large budded cells with divided nuclei) or in unbudded G1 cells (Figure 4C). The Cdc6-GFP signal was undetectable in the cells with small to large buds, confirming tight regulation of Cdc6 abundance by rapid degradation after S-phase onset. In sharp contrast, Cdc6-GFP was constitutively found in the nucleus throughout the cell cycle in *mck1* deletion cells (Figure 4C). This localization analysis was consistent with Western blot results that Cdc6p is stabilized in *mck1* deletion cells during S-phase and mitosis, as shown in Figure 4B and 4D.

**Figure 5:**
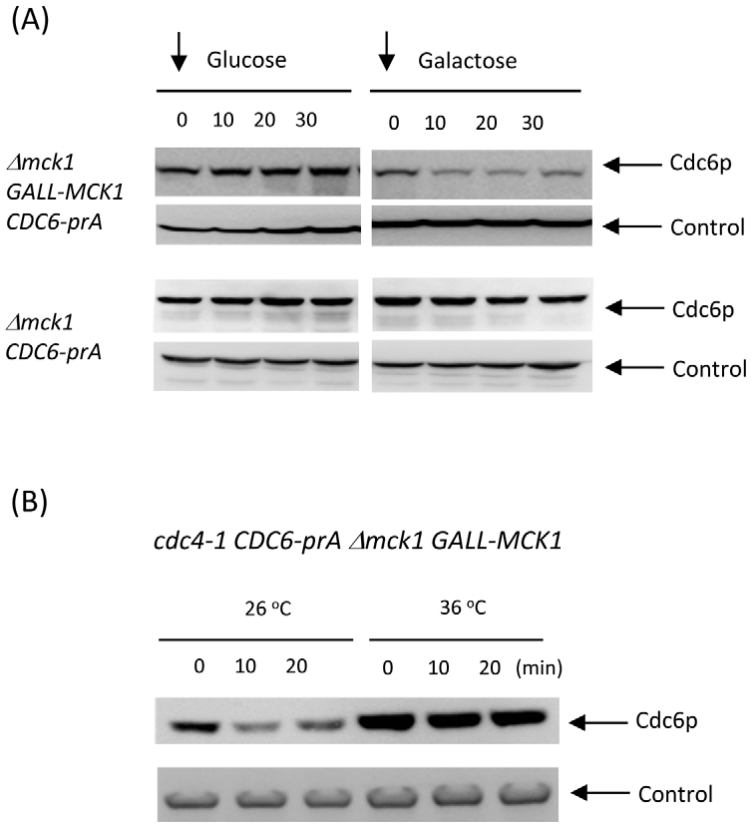
Rapid degradation of Cdc6p by Mck1p overexpression was inhibited in *cdc4* mutant. (A) First, the *Δmck1 GALL-MCK1 CDC6-prA* cells were incubated in glucose plus nocodazole in order to arrest the cell cycle during mitosis. Then, the nocodazole was removed and the media was switched to either glucose or galactose. Samples were taken every 10 minutes, proteins were extracted and subjected to western blotting as described above. Control experiments were performed using *Δmck1 CDC6-prA* cells to show that galactose does not affect Cdc6 level. Pgk1 was used as a loading control. (B) *cdc4-1 CDC6-prA Δmck1 GALL-MCK1* strain was incubated in raffinose-containing media first. The cell cycle was blocked by nocodazole and then the cells were further incubated either at 26°C or 36°C for 1.5 hours. Galactose was added to the media, samples were collected every 10 minutes and the protein extracts were subjected to western blotting to detect Cdc6-prA. Pgk1 was used as a loading control.

We also tested if overexpression of Mck1 promotes rapid Cdc6p degradation. Exogenously expressed Mck1p under the *GALL* promoter significantly reduced Cdc6p protein levels 10 minutes after the addition of galactose (Figure 5A, top right). This result supports the idea that Mck1p promotes Cdc6p degradation.

### Mck1-mediated Cdc6 degradation was inhibited in *cdc4-1* mutant

We next examined if Mck1-mediated Cdc6 degradation is due to SCF^CDC4^ ubiquitin ligase. When *cdc4-1 CDC6-prA mck1 GALL-MCK1* strain was incubated at 26 degrees, Cdc6p was rapidly degraded followed by galactose addition (Figure 5B). This is consistent with results in Figure 5A. When Cdc4 was inactivated at 36 degrees, Cdc6 became stable and was not degraded even after Mck1 overexperssion (Figure 5B). This result suggests that Mck1p phosphorylates Cdc6p to be subsequently recognized by SCF^CDC4^ complex for degradation.

### Mck1p binds to Cdc6p through a GSK-3 consensus site in the C-terminal region

**Figure 6:**
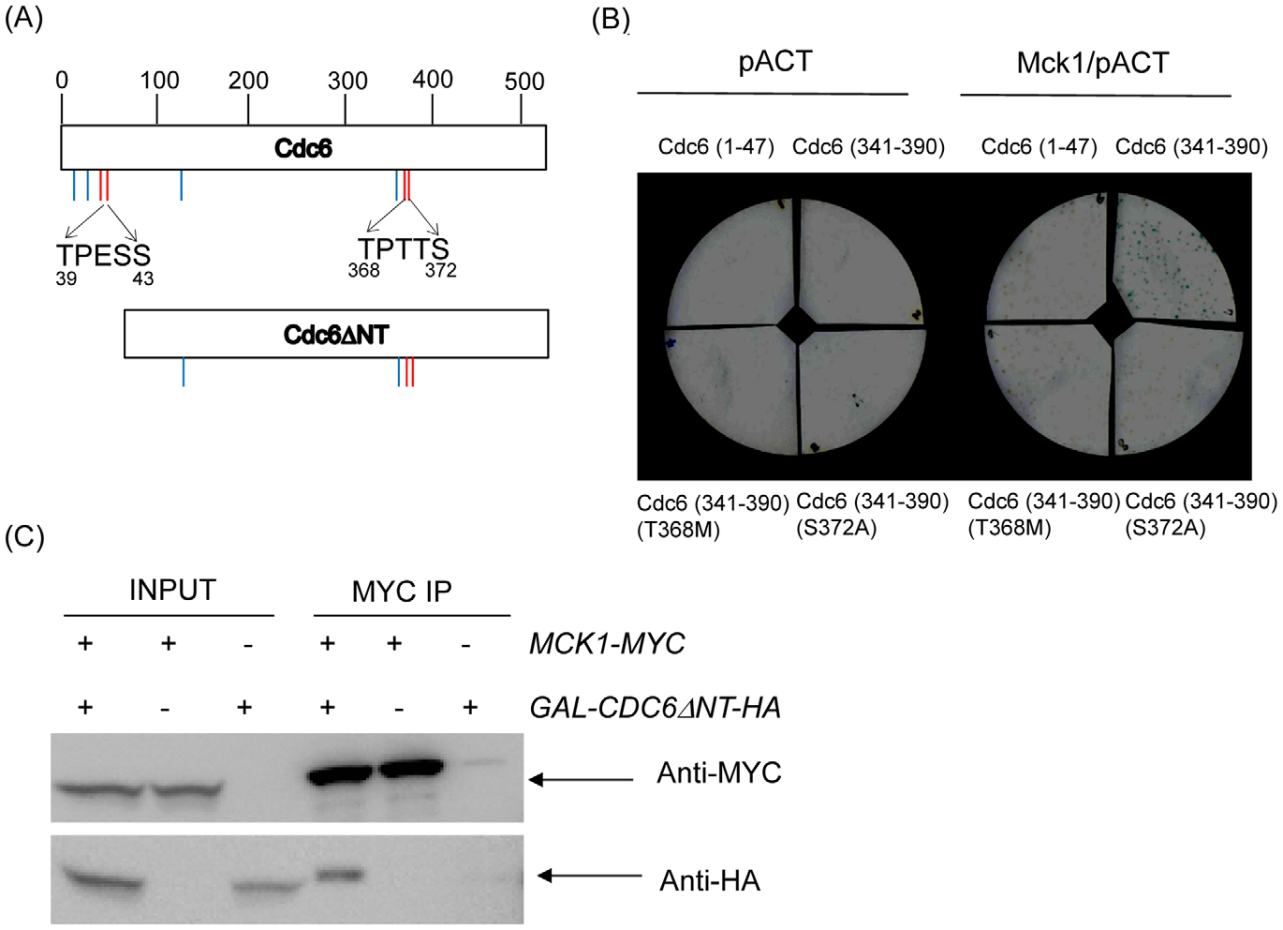
Mck1p interacts with Cdc6p through the GSK-3 consensus site in the C-terminal region. (A) Cdc6 contains eight CDK consensus sites, ^S^/_T_P motif indicated by short lines (top). The Cdc6ΔNT mutant lacks the N-terminal region, from amino acid 2–49, thereby lacking the first four CDK sites (bottom). The GSK-3 consensus sites located at 39–43 and 368–372 are indicated by short red lines. (B) Yeast two hybrid analysis was performed to determine the binding site between Mck1p and Cdc6p. A full length *MCK1* in pACT plasmid, containing the GAL4 activation domain (GAD), was co-transformed into L40 yeast strains along with the various *CDC6* mutants in the pBTM116 plasmid fused to LexA. Transformants were assayed for β-galactosidase activity as visualized in blue. (C) Co-immunoprecipitation analysis was performed using *MCK1-MYC CDC6ΔNT-HA*, *MCK1-MYC or CDC6ΔNT-HA* strain. Cells were incubated in raffinose to log phase and then switched to Galactose to induce *GAL-CDC6ΔNT*. After 2 hour incubation, nocodazole was added to the media in order to block cell cycle during G2/M. The cells were incubated for two more hours and protein was extracted. The protein lysate from each strain was incubated with agarose beads conjugated with anti-MYC to pull down the Mck1 complex. The protein complex was subjected to western blotting to analyze Mck1-MYC or Cdc6DNT-HA using anti-MYC or anti-HA antibodies, respectively. 1/50 of the original protein lysate was used as INPUT.

GSK-3 kinases phosphorylate the first serine or threonine residues in the consensus site followed by a phospho-serine or phospho-threonine at the position +4 [S/T-XXX-pS/T] [36]. There are two potential GSK-3 consensus phosphorylation sites in Cdc6p, TPESS (39–43) and TPTTS (368–372) (Figure 6A). To test if Mck1p binds Cdc6p at the GSK-3 consensus sites, we performed a yeast two-hybrid assay. We examined whether Mck1p, fused with Gal4 activation domain (GAD), interacts with various truncated *CDC6* mutants fused to the LexA DNA binding domain. Mck1p interacted with the C-terminal region of Cdc6p (aa341–390) and not with the N-terminus (aa 1–47) (Figure 6B). The mutation at T368M or S372A abolished two-hybrid interaction between Mck1p-Cdc6p indicating that Mck1p targets Cdc6p through the GSK consensus site at 368–372 (Figure 6B). The physical interaction between Mck1p and Cdc6p was also confirmed by co-immunoprecipitation (Co-IP) assay using the *MCK1-MYC GAL-CDC6ΔNT-HA* strain. Mck1p interacted with Cdc6ΔNTp, indicating that Mck1p interacts with Cdc6p, and the protein interaction was mediated through the C-terminal region in Cdc6p (Figure 6C). The protein binding between Mck1p and Cdc6p was observed only in mitotic arrested cells blocked by nocodazole and not in asynchronous culture or G1-arrested cells (data not shown). Therefore the physical interaction between Mck1p and Cdc6p is likely primed by mitotic CDK phosphorylation of the S372 site (see next section). We also noticed that Cdc6ΔNT migrates slower in the co-IP samples than the input, consistent with the idea that only the phosphorylated form of Cdc6, probably targeted by CDK, binds to Mck1 (Figure 6C).

### *CDC6* mutations that abrogate GSK-3 binding are lethal with the *orc* mutants

**Figure 7:**
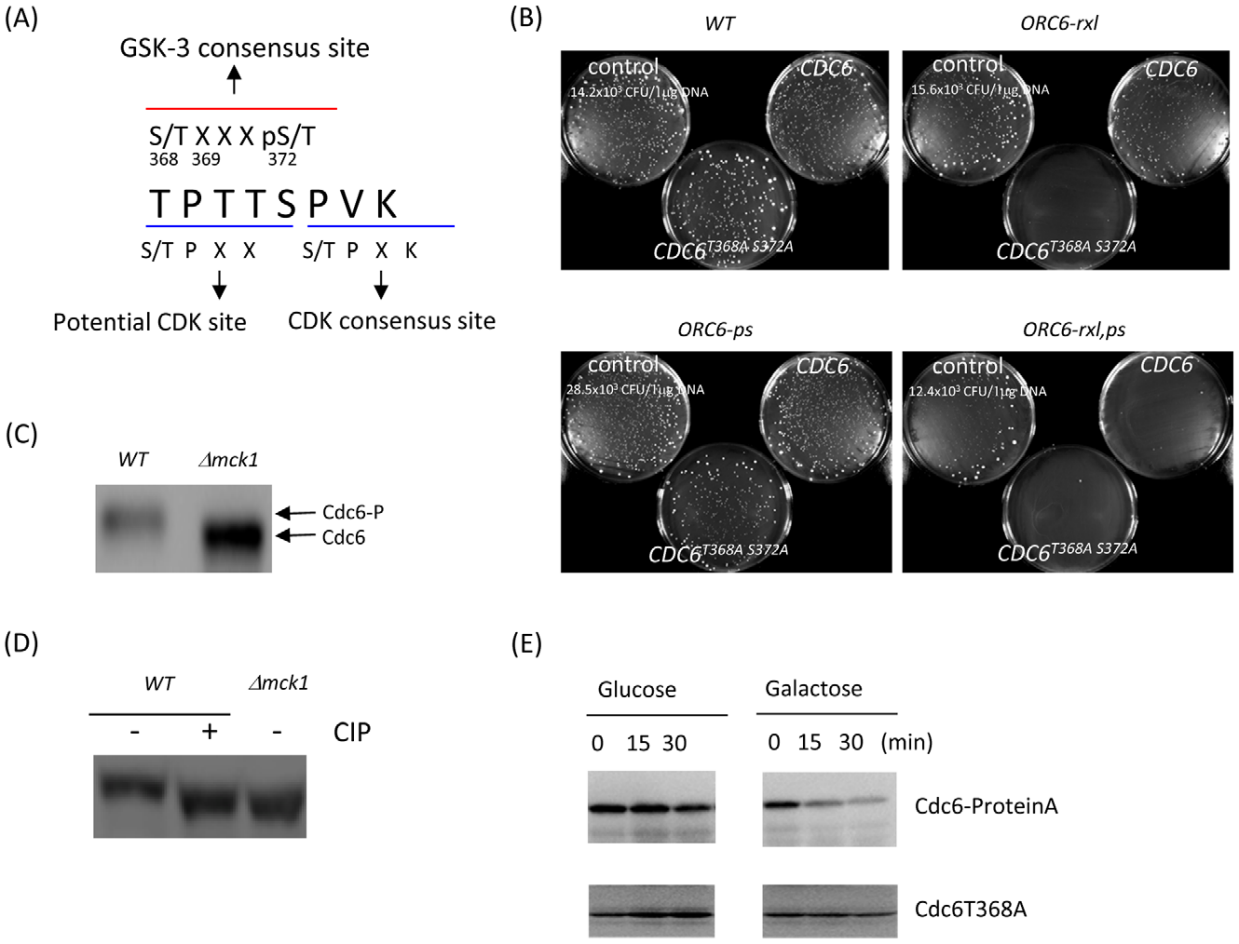
Mutations at the GSK-3 consensus site in Cdc6 play a role in Cdc6p stability. (A) GSK-3 and CDK consensus sites in the Cdc6 C-terminal region were shown as a red and blue line, respectively. (B) The *pRS426* (control plasmid), *CDC6* or *CDC6^T368A S372A^* in 2micron plasmids were transformed into *ORC6-WT, ORC6-rxl, ORC6-ps* or *ORC6-rxl,ps* mutants and plated on SD-Ura plates. Transformation efficiency for each strain was shown as CFU/µg DNA. (C) *Δmck1 cdc4-1 CDC6-prA* cells (labeled as *Δmck1*) or *cdc4-1 CDC6-prA* cells (labeled as *WT*) were arrested during metaphase by nocodazole for 1 hour and then temperature shifted to 37°C for 1.5 hour. Proteins were extracted and subjected to western blotting to detect Cdc6-prA. (D) Protein extracts in Figure 7C were treated with 10 units of CIP and incubated for 10 minutes at 4 degrees. 20 µl of protein sample from *Δmck1* cells or 60 µl of protein samples from WT cells were used in order to normalize the Cdc6 protein expression. (E) An extra copy of *CDC6* or *CDC6T368*A was integrated into a Δ*mck1 GALL-MCK1 CDC6-proteinA* strain at the URA locus. The resulting Δ*mck1 GALL-MCK1 CDC6-proteinA URA::CDC6T368A* cells were treated with nocodazole. Next, the mitotic arrested cells were incubated in YEPG to overexpress Mck1p and supplemented with nocodazole. Cells were collected every 15 minutes. Endogenous Cdc6-proteinA or exogenous Cdc6T368A was analyzed by western blot.

A GSK-3 kinase usually requires priming [36]. In Cdc6, the predicted priming site is located at S372 based on the amino acid sequence. After priming, the GSK-3 kinase phosphorylates the target site at the first serine or threonine that corresponds to T368 (see discussion). Next, we tested to see if mutations at the GSK-3 consensus phosphorylation site in *CDC6* cause lethality in *orc* mutants like the *mck1* deletion does. To prove that the C-terminus GSK-3 consensus site 368–372 in *CDC6* was involved in the inhibition of DNA re-replication, the potential phosphorylation site (T368) and the priming phosphorylation site (S372) were altered to alanine. The *CDC6-T368A S372A* in a 2 micron plasmid was transformed into wild type, *ORC6-rxl*, *ORC6-ps* or *ORC6-rxl,ps* mutants. Colonies formed when either *CDC6* wild type or *CDC6 T368A S372A* plasmids were transformed into the *ORC6*-wild type strain (Figure 7B, top left). In contrast, the *CDC6 T368A S372A* plasmid (but not *CDC6-wt*) was toxic in the *ORC6-rxl* cells, as transformants gave very few visible colonies (Figure 7B, top right). This effect was even more pronounced in *ORC6-rxl,ps* cells and, in this case, even the *CDC6-wt* plasmid appeared somewhat toxic (Figure 7B, bottom right). The *CDC6-T368A S372A* plasmid did not induce toxicity in the *ORC6-ps* cells (Figure 7B, bottom left) which confirmed the result that *mck1* did not genetically interact with *ORC6-ps* mutation (Figure 1). The plasmid harboring *CDC6-T368A* or *CDC6-S372A* single mutation was also toxic in the *ORC6-rxl* strain (Figure S2). These results suggest that the interaction of Cdc6p with Mck1p and/or its phosphorylation by Mck1p contributes to the down-regulation of Cdc6p levels.

### Mck1p phosphorylates Cdc6 for its degradation

To confirm that Cdc6p is phosphorylated by Mck1 *in vivo*, we analyzed the Mck1-dependent mobility shift of Cdc6p in the *cdc4-1* mutant background by western blot. We used *cdc4-1* mutant to prevent degradation of phosphorylated Cdc6 and examined the effect of Mck1 on the phosphorylation status of Cdc6p. Cdc6p in the wild type cells migrated slower that that in the *Δmck1* deletion cells indicating that Cdc6p is hyper-phosphorylated in wild type cells. (Figure 7C). In the *mck1* deletion cells, the signal of the higher molecular weight band was abrogated and the lower band was abundant suggesting that Cdc6p is less phosphorylated and more stable (Figure 7C right). To confirm that the slow migrating band of Cdc6p in the wild type cells is due to phosphorylation, protein extracts from wild type cells were treated with CIP (calf intestine phosphatase). After the CIP treatment, the slower migrating band of Cdc6p disappeared and the faster-migrating band was observed at the same level as that in *Δmck1* cells. It suggests that the band shift between wild type and *Δmck1* is due to phosphorylation (Figure 7C and 7D).

Finally we tested if Mck1p dependent destabilization of the Cdc6p is mediated by the T368 residue. The *mck1 GALL-MCK1 CDC6-proteinA CDC6T368A* strain contains both wild type Cdc6 (tagged with protein A) and Cdc6T368A (no tag). First the cells were arrested in mitosis with nocodazole and then released into galactose to overexpress Mck1p. Wild type Cdc6p was degraded rapidly after Mck1p overexpression, which is consistent with previous results in Figure 5A (Figure 7E, upper panel). In contrast, Cdc6T368A protein was resistant to degradation and was stable even after Mck1p overexpression (Figure 7E, lower panel). We also observed faster migration of Cdc6T368A protein than the wild type Cdc6p by western blot (Figure S3). We conclude that Cdc6p is phosphorylated at T368 by Mck1p to induce its degradation.

## Discussion

In this study, we show that a GSK-3-like kinase, Mck1p, is involved in the inhibition of DNA re-replication through its role in Cdc6p turnover in *S. cerevisiae*. There are 8 CDK consensus sites in *CDC6*. The first 47 amino acids at the N-terminus of Cdc6 are targeted by Cyclin/CDK and are critical for SCF^cdc4^ dependent proteolysis [7]. Stabilization of Cdc6p in *mck1* deletion cells suggests that CDK-dependent phosphorylation at the N-terminus of Cdc6 is not sufficient enough for CDC6p degradation *in vivo*, that Mck1-dependent phosphorylation through T368 site is also required. The Cdc6 T368A mutant was resistant to Mck1p-dependent degradation (Figure 7E). Nocodazole was added to the media throughout this experiment, therefore Cdc6 stabilization by the T368A mutation, even after Mck1p overexpression, is not due to a change in cell cycle progression. This is of particular interest because activation of CDK promotes both DNA replication and Cdc6p degradation at the same time. The requirement of Mck1 for Cdc6p degradation most likely ensures that degradation of Cdc6p occurs only after origin firing has been initiated.

Three distinct Cdc6p degradation modes have been proposed by Diffley's group [32]. Mode1 degradation during G1 phase is independent of Cdc6 CDK consensus sites and is mediated neither by SCF nor APC. The Cdc6p degradation by Mode 2 and Mode 3 are triggered later during the cell cycle. Mode3 is required for Cdc6 degradation during mitosis. The Cdc6p degradation by Mck1p accounts for the mode3 mechanism based on the Cdc6p stabilization pattern during mitosis in *mck1* deletion (Figure 4A). Diffley's group has reported that the Cdc6 T368M mutation leads to Cdc6p stabilization during mitosis and the mutation is resistant to mode 3 proteolysis by SCF^cdc4^ complex [6]. In this study, we showed that Mck1-dependent Cdc6 phosphorylation is targeted by SCF^CDC4^ complex for degradation (Figure 5B). Therefore, Mck1, most likely, phosphorylates Cdc6 and the phosphorylation at T368 is recognized by Cdc4. It is not clear if mode 3 requires CDK activity. Therefore Mck1p may promote complete Cdc6 degradation during mitosis in addition to its degradation mechanism through CDK phosphorylation. Further studies are required to test if Mck1 could also promote Cdc6 degradation via Mode 1 or Mode 2.

There are two potential GSK-3 sites S/TXXXpS/T in Cdc6, at 39-43 and 368-372 amino acid residues. It has been reported that these sites share sequence similarities and are targeted for SCF^CDC4^ dependent proteolysis [6]. Our yeast-two hybrid assay showed a specific interaction between Mck1p and Cdc6p through the GSK-3 consensus site located at residues 368–372 (TPTTS). This GSK-3 site in Cdc6p, amino acid 368–372, is also shared by two potential CDK phosphorylation sites 368–371 (TPTT) and 372–275 (SPVK). The former partially matches with a minimal consensus CDK phosphorylation site (S/T-P) whereas the latter perfectly matches an optimal CDK site, with a basic residue at the +3 position. It is important to note that Cdc6 is a very good substrate of the B-type Cyclin/CDK complex [37]. The GSK-3 kinase and CDK could share substrate specificity [38]. GSK-3 kinases require “priming” phosphorylation by another kinase on their substrates [36]. The priming site is usually located C-terminally of the GSK-3 phosphorylation site, at the +4 position, which corresponds to S372 in Cdc6. After priming, GSK-3 recognizes its target and can phosphorylate the first serine or threonine residue, which corresponds to T368 in Cdc6. Thus, C-terminal Cdc6p (aa 341–390), including the GSK-3 consensus phosphorylation sequence, is sufficient for Mck1 binding and their interaction likely depends on phosphorylation of S372 by CDK (Figure 6B). We propose a model in which S372 is phosphorylated by cyclin/CDK first in order to induce phosphorylation at T368 by Mck1p kinase. This priming model allows Cdc6 to create Cdc4 diphospho-degrons which is an efficient Cdc4 recognition site. David Morgan's group shows that Eco1 is primed by CDK and DDK in order to be targeted by Mck1, which creates Cdc4 recognition site (personal communication). Mck1 is involved in the degradation of SCF^CDC4^ substrates such as Rcn1and Hsl1 [25], [26], [39]. Therefore, the priming model to create Cdc4 diphospho-degrons seems to be a universal mechanism to regulate protein degradation.

Mck1p protein levels are not cell cycle-regulated (data not shown) therefore Mck1 activation is not regulated by its own expression level. This result supports the idea of the priming hypothesis in which Mck1 can target its substrate, Cdc6p, only after Cdc6 is phosphorylated by cyclin/CDK in a cell cycle-dependent manner. Given the requirement of T368 for Mck1 dependent degradation of Cdc6, Mck1 most likely phosphorylates this residue directly *in vivo*. However, it is formally possible that Mck1 affects Cdc4 function other than Cdc6. We favor the model that Mck1 directly phosphorylates Cdc6 to promote Cdc4-dependent degradation based on our results in Figure 5B, Figure 7C and 7D. Whether or not SCF^CDC4^ or other targets such as Sic1 are also phosphorylated by Mck1 is an interesting future study.

The glycogen synthase kinase-3 (GSK-3) was originally identified as a kinase that inactivates glycogen synthase [40]. In higher eukaryotes, there are two isoforms, GSK-3α and GSK-3β, that regulate various cellular processes including Wnt signaling [41] and insulin signaling [42], [43]. The yeast homologue of GSK3, Mck1p, also has diverse biological functions (see introduction). This is the first evidence to show that Mck1p or any GSK-3 kinase controls DNA replication. Whether GSK-3 kinases contribute to the regulation of DNA replication at other targets should be investigated further.

## Materials and Methods

### SGA analysis

SGA analysis was performed as previously described [19], [20]. A query strain, *MATalpha ORC6-rxl::LEU2 mfa::MFA1pr-HIS3 trp1 ade2 can1 leu2 his3 lys2 ura3*, was placed on YEPD in rectangle plates. Then deletion mutant arrays (*MATa geneX::KanMX TRP1 ADE2 met15 leu2 ura3 his3*) were put on top of the query strains. The resulting diploid cells were sporulated on the plates containing 2% agar, 1% potassium acetate, 0.1% yeast extracts, 0.05% glucose, supplemented with uracil and histidine. After incubation at 22 degrees for 5 days, the spores were pinned onto haploid selection plates (SD-His/Leu/Arg plus canavanine) to select for MATa *mfa*::*MFA1pr-HIS3 ORC6-rxl*::*LEU2* progeny, followed by pinning onto YEPD plates containing G418 to select out the deletion array mutants. Finally, double mutants were placed on SD-His/Leu/Arg plus canavanine plus G418 for 2 days. The proliferation of those that contained haploid cells was scored visually. The deletion sets used in this study were obtained from EuroScarf and are derivatives of BY4741 [44].

### Cell cycle blocks

First, *GAL-CDC6-HA* or *mck1 GAL-CDC6-HA* strains were grown in raffinose-containing media and then galactose was added to express Cdc6-HA for 2 hours. The cell cycle was blocked during mitosis by nocodazole at the concentration of 15 µg/ml for 2 hours. Next, glucose was added to the media to shut off the GAL expression (Figure 4A). *CDC6-PRA* or *mck1 CDC6-PRA* strains were grown in liquid YEPD to log-phase at 30 degrees and then treated with alpha-factor at the concentration of 100 nM for 2 hours. The cells were washed with YEPD three times to release the cell cycle from G1. Samples were collected every 10 minutes for 80 minutes for Figure 4B. To block the cell cycle during mitosis, *CDC6-PRA* or *mck1 CDC6-PRA* strains were treated with nocodazole at the concentration of 15 µg/ml for 2.5 hours at 30 degrees. The mitotic block was released by washing cells with YEPD twice. Samples were collected every 10 minutes for 60 minutes for Figure 4D. For Figure 5 and 7D, cells were treated with nocodazole for 2 hours and then switched to YEPD or YEPG containing nocodazole at 15 µg/ml.

### Plasmids and strains

All strains used, except for SGA analysis, are derivatives of W303 (strain list in Table S1). Standard methods were used for mating and tetrad analysis. DNA transformation was performed by the lithium acetate method [45]. To generate *mck1* or *mre11* deletion in the W303 background, genes disrupted by a *KanMX* cassette in BY4741 haploid deletion libraries (EuroScarf) were amplified by PCR. The PCR product containg the *KanMX* cassette with *MCK1* flanking region was transformed into the wild type W303 strain. The resulting *mck1* deletion cells in W303 were confirmed by PCR. The *MCK1-MYC* strain was generated by PCR genomic integration of a PCR product containing a *MYC* tag and a *TRP* gene [46]. *GAL-CDC6ΔNT-HA* strain and plasmid were kindly provided by Dr. Stephen Bell. The *ORC6-rxl*, *ORC6-ps*, *ORC2-ps* and *MCM7-NLS* mutations were described previously [11], [13], [34]. *CDC6-proteinA* strain was generated as previously described [47]. *Rad53-FLAG* strain was obtained from Dr. Petrini [48]. To generate *GALL-MCK1*, *MCK1* gene was cloned into *GALL-pRS405* plasmid at BamHI and SpeI sites using *MCK1* plasmid provided by Dr. P. Hieter [28]. The resulting *GALL-MCK1/pRS405* plasmid was cut with BstEII, and the linearized plasmid was transformed into *bar1 mck1::KanMX CDC6-prA::HIS3* strain to integrate *GALL-MCK1* at *LEU* locus. Cdc6-GFP strain was made by direct transformation of a GFP cassette [49] in BY4741 and subsequently back crossed to W303-1B three times for Figure 4C. *CDC6* plasmid was generated by PCR method using W303 wild type genomic DNA. The resulting PCR product was cloned into pYES2.1 Topo TA plasmid (Invitrogen) for Figure 7B. The CDC6/pYES2.1 plasmid was subjected to site-directed mutagenesis using QuickChange Site-directed mutagenesis kit (Agilent Technologies, CA) to introduce T368A S372A mutation for Figure 7B. *CDC6/pRS406* plasmid was generated by PCR cloning. *CDC6* gene including the endogeneous promoter (300 bp upstream from the start codon) was amplified by PCR using primers that contain BamHI and XhoI, and cloned into *pRS406* at BamH1and XhoI sites. The *CDC6/pRS406* plasmid was used as a temperate to generate *CDC6-T368A/pRS406*. Site-directed mutagenesis was performed as described above. The resulting plasmids were cut with NcoI to integrate the mutated *CDC6* at *URA3* locus in *mck1 GALL-MCK1 CDC6-proteinA* strain for Figure 7E.

### Making a temperature-sensitive mutant of *mck1*

A temperature sensitive mutant of *MCK1* was generated using a previously described method [34]. *MCK1* gene was cloned into *pRS414* at BamHI and SpeI sites from *MCK1* plasmid provided by Dr. P. Hieter [28]. The *MCK1/pRS414* plasmid was mutagenized by PCR mutagenesis to introduce random mutations in the *MCK1* gene as previously described [50]. The mutagenized *mck1/pRS414* plasmid was transformed into *mck1 orc6-rxl* strain containing *MCK1/pRS416* plasmid. The *mck1 orc6-rxl* cells containing mutagenized *mck1* plasmid were tested for its viability at 37 degrees. The mutagenized *mck1/pRS414* plasmid (*mck1-16* mutation) was isolated from the strain and was inserted into the *pRS406* plasmid at BamHI SpeI sites. The resulting *mck1-16/pRS406* plasmid was cut with BstEII restriction enzyme and was integrated at the *URA3* genome locus. Sequence analysis identified two mutations in the temperature sensitive *mck1-16* allele, resulting in P275L and E357G.

### Co-IP and Western blotting

A 50-ml culture of each strain was grown to log-phase an OD595 of 0.5 was reached. The cell pellets were washed in cold TE buffer, and resuspended with 400 µl of protein extraction buffer [20 mM HEPES, pH 7.4, 110 mM potassium acetate, 2 mM MgCl_2_, 0.1% Tween 20, 1 mM DTT, 2 µg/ml DNaseI, protease inhibitor cocktail (Sigma-Aldrich, MO) and phosphatase inhibitor (Sigma-Aldrich, MO)]. Acid-washed glass beads (0.15 g) were added, and cells were disrupted by FastPrep (MP Biomedicals, OH) for 20 seconds, twice, at speed 6. Samples were centrifuged and 10 µl of supernatants were kept for Western blotting as “INPUT”. The remaining protein extracts were subjected to co-immunoprecipitation (Co-IP). Agarose beads conjugated with anti-MYC antibody (A7470) (Sigma-Aldrich, MO) were pre-incubated with 5% BSA in protein extraction buffer for 1 hour at 4 degrees to reduce non-specific binding first. Then the beads were mixed with the protein extract supernatants and rotated for 2 hours at 4 degrees. Beads were washed with protein extraction buffer five times. After the final wash, 30 µl of 2× sample buffer was added to the beads, and the protein was denatured at 95 degrees for 5 minutes. Proteins were separated by SDS-PAGE with Novex 4–20% Tris-Glycine polyacrylamide gel (Invitrogen, CA) except Figure 7C with 7% acrylamide large gel. The proteins on the gels were transferred to PVDF membrane (Millipore, MA). Western blot analysis was performed using anti-MYC antibody 9E10 (M4439) (Sigma-Aldrich, MO) at 1∶4000 dilution, anti-HA antibody 3F10 (Roche, IN) at 1∶4000 dilution and anti-FLAG antibody (A8592) (Sigma-Aldrich, MO) at 1∶4000 dilution. Cdc6-proteinA was visualized using anti-peroxidase soluble complex antibody produced in rabbit (P1291) (Sigma-Aldrich, MO) at 1∶4000 dilution. Cdc6 was detected using anti-Cdc6 antibody (9H8/5) (Abcam, MA) at 1∶500 dilution.

### Fluorescence microscopy

Log phase cultures of Cdc6-GFP expressing cells in SC medium supplemented with 20 mg/L adenine were imaged live with an Eclipse E600 fluorescence microscope (Nikon) equipped with a DC350F CCD camera (Andor) and 100×, NA 1.45, or 60×, NA 1.4, oil objectives. The images were captured with NIS-Elements software (Nikon) and prepared using Photoshop software.

### Flow cytometry

DNA content analysis by FACScanto (BD Biosciences, NJ) was performed as described previously [51].

### Yeast two-hybrid assay

The pBTM116 constructs containing various Cdc6 mutants were obtained from Dr. J. Diffley's lab [6]. Full length *MCK1* was cloned into pACT at BamHI and XhoI sites by PCR method. The *MCK1*/pACT and each of the various *CDC6*/pBMT116 plasmids were co-transformed into L40 strain and plated on SD-Leu/Trp plates [52]. The colonies were transferred to nitrocellulose membrane and kept at −80 degrees overnight. The membrane was placed on whatman paper soaked with 3 ml of Z buffer, [60 mM Na_2_HPO_4_, 40 mM NaH_2_PO_4_, 10 mM KCl, 1 mM MgSO_4_] with 300 µg/ml X-gal and 0.044 M 2-mercaptoethanol. The membrane was incubated at 30 degree overnight to visualize the blue colonies.

## Supporting Information

Figure S1 *CDC6-prA* or *Δmck1 CDC6-prA* strains were incubated in YEPD and cell cycle arrested during mitosis using nocodazole. CHX at the concentration of 100 ug/ml was added to the media and samples were collected every 20 minutes. Cdc6-prA levels were quantified and shown as a bar graph.
(TIF)

Figure S2 Genetic interactions between various *CDC6* mutants and *ORC6-rxl*. *CDC6T368A, P369A, S372A, P373A* or *T368A S372A* in 2μ plasmids were transformed into either wild type or *ORC6-rxl* strains, and plated on YEPD plates.
(TIF)

Figure S3 Cells were treated with nocodazole first, and the temperature was shifted to 36 degrees in order to inactivate Cdc4 function. Proteins were extracted and subjected to western blot using direct antibody against Cdc6.
(PPT)

Table S1 Genotype of all strains used in the study. All strains have W303 genetic background.
(PDF)
