# Supplementary figures and images for: A Yeast GSK-3 Kinase Mck1 Promotes Cdc6 Degradation to Inhibit DNA Re-Replication
Source: PLoS Genet. 2012 Dec 6;8(12):e1003099. doi: 10.1371/journal.pgen.1003099 (PMC3516531; doi:10.1371/journal.pgen.1003099)

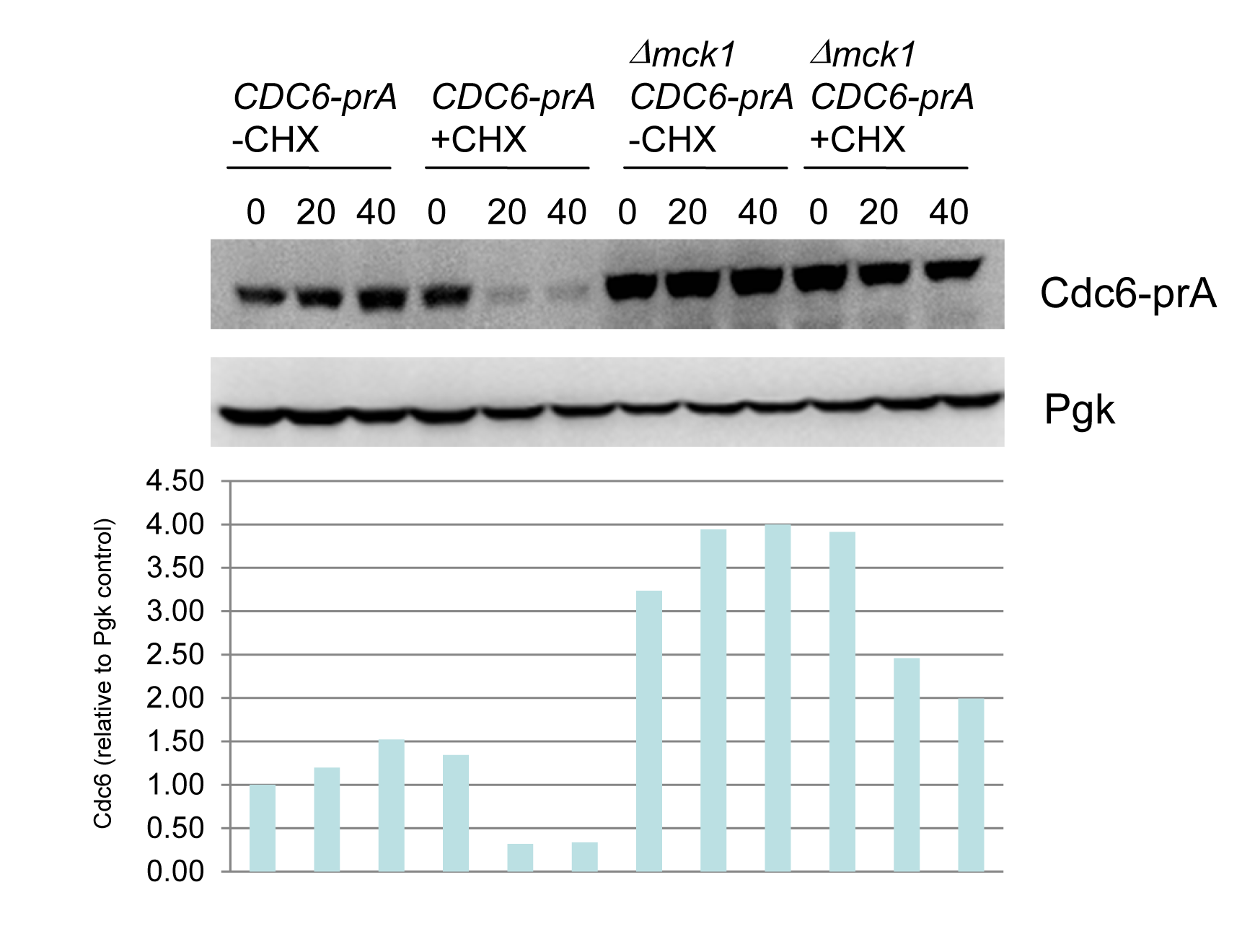

Supplement: Figure S1 — CDC6-prA or Δmck1 CDC6-prA strains were incubated in YEPD and cell cycle arrested during mitosis using nocodazole. CHX at the concentration of 100 ug/ml was added to the media and samples were collected every 20 minutes. Cdc6-prA levels were quantified and shown as a bar graph. (TIF) [file pgen.1003099.s001.tif]

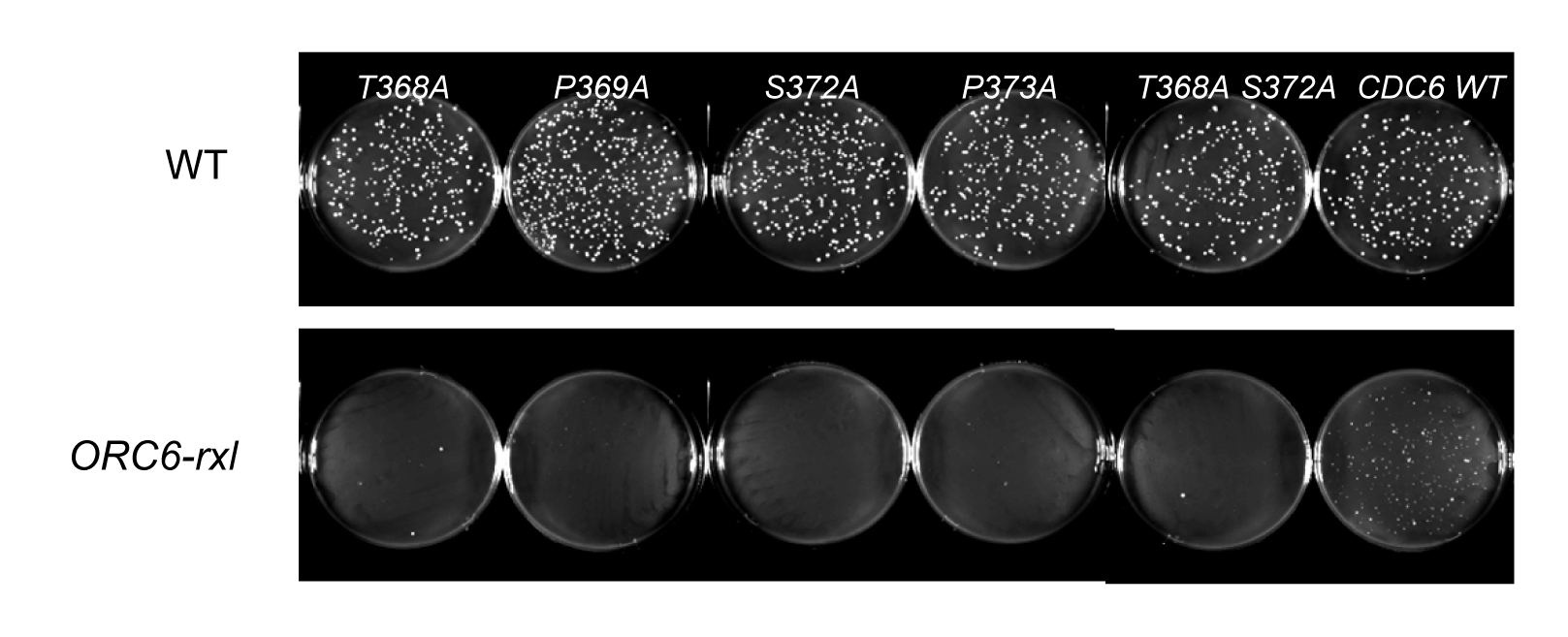

Supplement: Figure S2 — Genetic interactions between various CDC6 mutants and ORC6-rxl. CDC6T368A, P369A, S372A, P373A or T368A S372A in 2μ plasmids were transformed into either wild type or ORC6-rxl strains, and plated on YEPD plates. (TIF) [file pgen.1003099.s002.tif]

## Slide 1
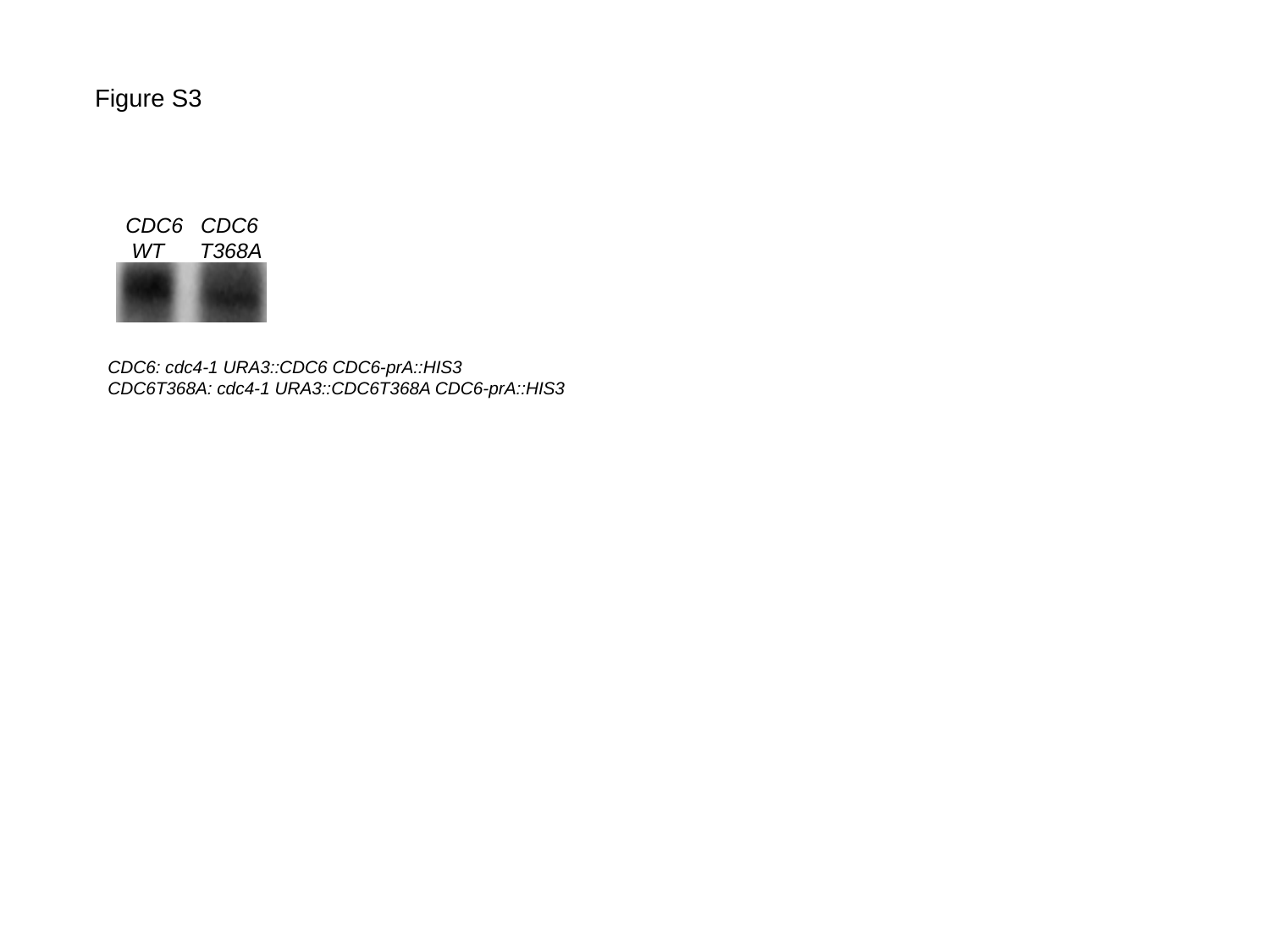

Figure S3
CDC6 CDC6
 WT T368A
CDC6: cdc4-1 URA3::CDC6 CDC6-prA::HIS3
CDC6T368A: cdc4-1 URA3::CDC6T368A CDC6-prA::HIS3

Supplement: Figure S3 — Cells were treated with nocodazole first, and the temperature was shifted to 36 degrees in order to inactivate Cdc4 function. Proteins were extracted and subjected to western blot using direct antibody against Cdc6. (PPT) [file pgen.1003099.s003.ppt]
