# Supplementary material for: A Yeast GSK-3 Kinase Mck1 Promotes Cdc6 Degradation to Inhibit DNA Re-Replication
Source: PLoS Genet. 2012 Dec 6;8(12):e1003099. doi: 10.1371/journal.pgen.1003099 (PMC3516531; doi:10.1371/journal.pgen.1003099)
Supplement: Table S1 — Genotype of all strains used in the study. All strains have W303 genetic background. (PDF) [file pgen.1003099.s004.pdf]

Table S1. Strain list

Strain Name	Genotypes	origin
W303	*leu2-3,112 trp1-1 can1-100 ura3-1 ade2-1 his3-11,15*	
RUY005	*MATa orc6::HISMX6::LEU2::ORC6-rxl lys2*	Stephen Bell (2004)
RUY006	*MATa orc6::HISMX6::LEU2::ORC6-ps lys2*	Stephen Bell (2004)
RUY156	*MATa ORC2-ps lys2 [ADE]*	Joachim Li
BCY037	*MATα mck1::KanMX*	This study
BCY002	*MATα orc6::HISMX6::LEU2::ORC6-rxl URA3::mck1-16 mck1::KanMX*	This study
BCY009	*MATα clb5::HIS3 URA3::mck1-16 mck1::KanMX*	This study
BCY259	*MATa URA3::mck1-16 mck1::KanMX ADE2*	This study
BCY051	*MATa mre11::KanMX orc6::HISMX6::LEU2::ORC6-rxl URA3::mck1-16 mck1::KanMX*	This study
BCY079	*MATα orc6::HISMX6::LEU2::ORC6-rxl,ps URA3::mck1-16 mck1::KanMX*	This study
BCY085	*MATa orc6::HISMX6::LEU2::ORC6-rxl,ps mck1-16 mck1::KanMX MCM7-NLS TRP::DDC2-GFP*	This study
BCY083	*MATa orc6::HISMX6::LEU2::ORC6-rxl,ps mck1-16 mck1::KanMX ORC2-ps*	This study
BCY089	*MATα orc6::HISMX6::LEU2::ORC6-rxl,ps mck1-16 mck1::KanMX MCM7-NLS ORC2-ps TRP::DDC2-GFP*	This study (*MCM7-NLS* from Li's lab)
BCY306	*MATα LEU2::ORC6-rxl,ps MCM7-NLS*	This study
BCY308	*MATα LEU2::ORC6-rxl,ps ORC2-ps*	This study
BCY307	*MATa LEU2::ORC6-rxl,ps MCM7-NLS ORC2-ps*	This study
BCY107	*MATα RAD53-FLAG orc6::HISMX6::LEU2::ORC6-rxl mck1-16 mck1::KanMX*	This study (*RAD53-FLAG* is from Petrini's lab)
BCY252	*MATa bar1 URA3::GAL-CDC6-HA(s) ADE2*	This study
BCY254	*MATa bar1 mck1::KanMX URA3::GAL-CDC6-HA(s) ADE2*	This study
BCY077	*MATa bar1 HIS3::CDC6-ProteinA*	This study (*HIS3::CDC6-ProteinA*

		is from Fred Cross (2003))
BCY078	*MATa bar1 HIS3::CDC6-ProteinA mck1::KanMX*	This study
BCY223	*MATa CDC6-GFP-HIS3*	This study
BCY224	*MATa mck1::KanMX CDC6-GFP-HIS3*	This study
BCY133	*MATa bar1 HIS3::CDC6-ProteinA mck1::KanMX LEU2::GALL-MCK1*	This study
BCY309	*MATa cdc4-1 mck1::KanMX CDC6-prA::HIS3 LEU2::GALL-MCK1 ADE*	This study
BCY103	*MATa TRP1::MCK1-9MYC URA3::GAL-CDC6ΔNT-HA*	This study
BCY105	*MATa bar1 TRP1::MCK1-9MYC*	This study
BCY282	*MATa cdc4-1 CDC6-prA::HIS3 ADE*	This study
BCY284	*MATa cdc4-1 mck1::KanMX CDC6-prA::HIS3 ADE*	This study
BCY221	*MATa bar1 mck1::KanMX CDC6-prA::HIS3 LEU2::GALL-MCK1 URA::CDC6T368A*	This study
